# Editorial: Enhancing plant resilience and productivity through biostimulants and advanced biotechnological approaches

**DOI:** 10.3389/fpls.2026.1864212

**Published:** 2026-05-06

**Authors:** David W. M. Leung, Boon Chin Tan

**Affiliations:** 1 School of Biological Sciences, University of Canterbury, Christchurch, New Zealand; 2 Centre for Research in Biotechnology for Agriculture, University of Malaya, Kuala Lumpur, Malaysia

**Keywords:** genome editing, nematode, oxidative stress, ozone, phytophthora, plant transformation, salinity stress, soil amendments

Global food security depends heavily on sustainable crop growth and productivity, which are increasingly affected by multiple challenges, including climate change, plant pests, and diseases. Traditional plant breeding approaches to develop improved crop varieties with enhanced resilience and productivity have well-known limitations, as they are time-intensive, laborious, and costly. The aim of this Research Topic is to host research contributions using advanced biotechnological approaches, including novel biostimulants and molecular tools, to develop more efficient and sustainable solutions for safeguarding crop growth and productivity.

Naranjo et al. investigated the potential of protease-digested red winery waste products (lees) as a new biostimulant for crop protection against atmospheric ozone. In pot trials with grapevine (*Vitis vinifera*), an ozone-sensitive and economically important crop in Mediterranean countries, foliar application of lees reduced ozone-induced oxidative stress in photosynthetic parameters. This effect was accompanied by differential regulation of genes, including overexpression of genes associated with oxidative stress protection.

Aljeddani et al. evaluated the potential of sourcing valuable biostimulants from halophytes (plants tolerant of saline environments) to mitigate salinity stress. In their study, aqueous extracts were prepared from *Halocnemum strobilaceum*, a succulent plant that survives and germinates in the presence of up to 0.5 M NaCl. Foliar spray of the extracts was found to mitigate some of the negative impacts of high levels of NaCl on the growth and seed yield of *Chenopodium quinoa* (quinoa), a salinity-stress-sensitive, high-protein-yielding crop. In addition, the changes in seed protein compositions, including the induction of new proteins, were investigated.

Staykov et al. assessed the consistency of the foliar application of a commercial seaweed-based biostimulant on yield traits of pepper and eggplant under real agronomic conditions in a two-year study. Moreover, the metabolic, mineral and transcriptional gene changes that appeared to underpin the biostimulant’s promotion of fruit yield in both pepper and eggplant were uncovered.

Li et al. investigated the effects of soil amendment with oyster shell powder (a biological waste product) on improving the yield of peanut in marginal soils, such as acidic soils (red soils in China), over 5 years under field conditions. Various soil properties, including changes in soil pH and soil cadmium (Cd) bioavailability, were found to be critical for improving peanut yield.

Sellami et al. reported a systematic bibliometric evaluation of 222 studies published between 2000 and 2024 investigating the effects of biostimulants on wheat under field conditions. This consolidates the groundwork for ways forward to promote sustainable wheat farming through the use of environmentally friendly biostimulants.

Geremew et al. evaluated the biotechnological application of nanotechnology for the improvement of crop growth. In their study, zinc oxide nanoparticles (ZnO NPs) were used to prime *Amaranthus tricolor* (amaranth) seeds. The beneficial effects of ZnO NPs on the morphological and biochemical characteristics associated with the critical stage of early crop seedling establishment were investigated.

Danial et al. evaluated the genetic engineering strategy of heterologous constitutive expression of flowering pathway genes to enhance tomato fruit yield (increased fruit number). The transgenic tomato lines expressing *SUPPRESSOR OF OVEREXPRESSION OF CONSTANS 1* (*SOC1*) from soybean, maize and blueberry were evaluated for their effects on fruit productivity and other transcriptional changes.

Achary et al. reviewed new tools and approaches to accelerate the workflow for plant genetic engineering and genome editing across diverse crop varieties to address the challenges of improved crop productivity and food security. A snapshot of the recent discovery of a distinct set of plant regenerating peptides and morphoregulating genes is of great interest. In addition, the potential of the novel approach of genome editing to activate plant regeneration genes may also be of help to expedite improved crop variety development.

Liu et al. evaluated the suitability of a droplet digital PCR (ddPCR) assay for detecting plant pathogens, such as *Phytophthora nicotianae*, which can severely reduce crop productivity. It compares well with conventional qPCR in terms of accuracy and sensitivity for pathogen detection, but ddPCR involves less complex PCR optimization. This is of interest, as ddPCR has not been widely used for plant pathogen monitoring to improve crop management.

Wang et al. assessed a combined approach of priming with a biostimulant and gene transfer to enhance resistance to soybean cyst nematode (*Heterodera glycines* Ichinohe, SCN). Firstly, coating soybean seeds with a nematotoxic substance (cyclo Prol-Tyr) or CPT isolated from the bacterium Sneb545 enhanced nematode resistance in the roots of soybean seedlings. Secondly, as increased peroxidase activity and lignin content appeared to be associated with enhanced nematode resistance in CPT-primed soybean, a candidate gene (*GmPOD53L*) for enhanced nematode resistance was studied in a transient overexpression assay in soybean roots, demonstrating promising nematode resistance potential.

The studies in this Research Topic highlight the potential of combining biostimulants with advanced biotechnological tools to enhance crop resilience and productivity under growing environmental stresses. Diverse bio-based resources and innovative approaches, including nanotechnology, genetic engineering, genome editing, and improved pathogen detection, demonstrate promising, sustainable alternatives to conventional practices. Together, these contributions support a more integrated and efficient paradigm for crop improvement. Future efforts should prioritize field validation and scalability to translate these advances into practical solutions for strengthening global food security.

